# Understanding Australian health professionals’ knowledge of concussion: a cross-sectional nationwide survey

**DOI:** 10.1186/s12913-026-15122-9

**Published:** 2026-08-13

**Authors:** Sara L. Whittaker, Aleksandra K. Gozt, Lisa Licciardi, Naomi Fuller, Melinda Fitzgerald, Shenane Hogg, Natasha K. Brusco

**Affiliations:** 1 Rehabilitation, Ageing and Independent Living (RAIL) Research Centre, School of Primary and Allied Health Care, Monash University, Level 3, Building G, Peninsula Campus, McMahons Road, Frankston, VIC 3199 Australia; 2 Connectivity Traumatic Brain Injury Australia, Ralph and Patricia Sarich Neuroscience Research Institute, 8 Verdun Street, Nedlands, Western Australia 6009 Australia; 3 Level 4, Building G, Peninsula Campus, Department of Occupational Therapy, School of Primary and Allied Health Care, Monash University, McMahons Road, Frankston, VIC 3199 Australia; 4 Curtin Medical Research Institute, Curtin University, Bentley, WA 6102 Australia; 5 Perron Institute for Neurological and Translational Science, Nedlands, WA 6009 Australia

**Keywords:** Concussion, Health professionals, Doctors, Nurses, Physiotherapists, Occupational therapists, Mild traumatic brain injury, Persistent post-concussion symptoms

## Abstract

**Background:**

Concussion is a common condition following head injury affecting people across the lifespan. The purpose of this study was to understand the current state of concussion knowledge held by Australian health professionals, namely medical, nursing and allied health professionals. The primary aim was to establish health professionals’ knowledge of concussion, specifically its: (i) mechanisms of injury; (ii) symptoms; (iii) management; (iv) differential diagnosis; (v) consequences; and vi) to determine the current frequency and type of care provided to concussion patients.

**Methods:**

An electronic survey was conducted. Participants included Australian health professionals who had worked in a professional capacity in the last 12-months. Convenience sampling was used for nationwide data collection over a 9-month period disseminated via email (e.g. through professional associations) and social media, allowing for snowballing. Quantitative methods of analysis were primarily adopted, supplemented by qualitative content analysis for free text responses.

**Results:**

There were 126 participants (survey completion rate, 58%), who were most frequently female (*n* = 98/126, 78%), had more than 10 years of health professional experience (*n* = 88/126, 69%), and were aged between 30 and 39 years (*n* = 38/126, 30%). Participants were physiotherapists (*n* = 39/126, 31%), occupational therapists (*n* = 23/126, 18%), nurses (*n* = 17/126, 14%), medical professionals (including General Practitioners (GPs), physicians) (*n* = 13/126, 10%) and other health professionals (*n* = 20/126, 16%). Most participants correctly identified concussion injury mechanisms (*n* = 85/126, 68%); few participants correctly identified all concussion symptoms (*n* = 30/126, 24%); half the participants indicated they would frequently rely on published concussion management guidelines to inform practice (*n* = 65/126, 52%); differential diagnoses was considered by health professionals to varying degrees (e.g. intracranial injury (*n* = 57/64, 89%), and only some health professionals reported knowledge of persistent post-concussion symptoms (*n* = 78/126, 62%). The frequency and type of care provided to patients with concussion varied, but most frequently involved weekly (*n* = 25/66), or monthly (*n* = 10/66, 15%) reviews/treatment, with onward referral to Emergency Medicine Specialists being frequent for diagnosis of concussion (*n* = 11/17, 65%), and to a GP for treatment within 3 months (*n* = 7/18, 39%).

**Conclusions:**

While most Australian health professionals understand the mechanisms of injury for concussion, a gap exists regarding concussion symptoms, differential diagnoses, consequences and management. These gaps form a framework for future targeted education opportunities for health professionals who provide care to patients with a suspected or actual concussion.

**Supplementary Information:**

The online version contains supplementary material available at https://doi.org/10.1186/s12913-026-15122-9.

## Background

Concussion, a form of traumatic brain injury (TBI), is a common condition following an injury to the head or body, affecting people across the lifespan. For many people, concussion symptoms are transient, however, some may experience persistent symptoms requiring ongoing clinical care [1]. It is challenging to estimate the true incidence of concussion due to under reporting and varying definitions of concussion [2]. However, it is estimated that of the 69 million traumatic brain injuries sustained globally per year, of these 56 million are considered mild and classified as concussion [3]. In Australia, it is estimated that 170,000 cases of concussion occur per year, with only 20,000 presenting to an emergency department (ED), indicating that a majority of cases are managed in the community, outside of the hospital system, or treatment is not sought at all [4]. Clinical practice guidelines are essential for health professionals to deliver evidence-based care as they provide synthesised knowledge in a clear and concise format [5], yet clinical practice guidelines to manage concussion in the community, outside of sporting associations, have only just become available in Australia [6].

Globally, there is inconsistency in medical professionals’ concussion knowledge and management, with some evidence suggesting that new doctors receive very little concussion specific training [7–10]. In Australia, the national Australian and New Zealand (ANZ) Concussion Guidelines were recently released [11], however prior to this, Australian health professionals relied on international guidelines to inform their practice. Despite the release of the ANZ Concussion Guidelines, the implementation of evidence-based care into clinical practices can take a long time [12], often involving behaviour change, knowledge translation support strategies, as well as requiring health professionals to prioritise changes in clinical practice [13–15]. One strategy identified to support the implementation of clinical practice guidelines is providing targeted education to health professionals [16]. Targeted education ensures health professionals are aware of the condition, have knowledge of both the condition and best practice management of the condition, and understand how to translate the best practice management of the condition into clinical practice [5, 15, 16]. Considering this, the present study aimed to better understand the current ‘baseline’ of Australian health professionals’ awareness about concussion, including their knowledge and current clinical practice, to inform future education initiatives to enhance evidence-based concussion care in Australia. This included consideration of issues relevant to Aboriginal and Torres Strait Islander peoples, recognising persistent health inequities and barriers to accessing culturally safe care and the importance of addressing Indigenous health priorities in research [17].

The primary aim of this study was to establish Australian health professionals’ (medical, nursing and allied health professionals) current knowledge of concussion, specifically its: (i) mechanisms of injury; (ii) symptoms; (iii) management; (iv) differential diagnosis; (v) consequences; and vi) the current frequency and type of care (e.g., diagnosis, ongoing treatment) provided to patients with concussion. The secondary aim was to establish whether professional characteristics impact the health professional’s level of concussion knowledge.

## Methods

### Study design

This cross-sectional convergent mixed methods design with quantitative priority (QUAN+qual) [18] study was approved by Monash University Human Research Ethics Committee (ID: 42213), and adhered to the Declaration of Helsinki. This study has been reported in accordance with the Checklist for Reporting Results of Internet E-Surveys (CHERRIES) checklist [19].

### Setting and participants

The present study was conducted nationwide across Australia, inviting medical, nursing and allied health professionals to participate. To be eligible to participate in the study, participants were required to be over 18 years of age. Participants were not included in the study if they ceased working as a medical, nursing or allied health professional more than 1 year prior to survey completion. At the beginning of the survey, participants were advised about the purpose of the study, the anticipated completion time, and data storage details (i.e., duration of storage and where it will be stored). Following, consent was implied by the participant proceeding to complete the survey items and selecting to submit their responses at the conclusion of the survey. Written informed consent was obtained prior to survey completion. The survey did not require participant registration, and aggregated deidentified data.

A priori, the recruitment target was 200 participants, with a minimum of three health professions represented (e.g., medical, nursing, physiotherapy, occupational therapy, aboriginal health worker). Additional targets included 60 female participants (30% of *n* = 200 target sample); and 20 participants working in regional, remote or very remote communities (10% of *n* = 200 target sample). The a priori targets were set consistent with the expected demographic profile of health professionals within Australia. To monitor response rates and recruitment strategy throughout data collection, monthly steering committee meetings were held (including representation from the authorship group) to review proportions of respondents. Recruitment was initially planned for three months, with a provision to extend the recruitment period if recruitment was slow (i.e., if the a priori sample size was unlikely to be reached within a three-month period, or if the sample was not representative of the targeted population). The target of 200 participants was based on previous research examining GP concussion knowledge in Western Australia [7], combined with a pragmatic approach to a representative sample size.

### Data collection

The online survey informed by previously completed studies was developed on the Research Electronic Data capture (REDCap) data capture tool hosted and managed by Helix (Monash University) [4, 20–22]. Survey responses were entered by participants and stored on the REDCap database. Data collection occurred from July 2024 to March 2025 (9 months, extended due to slow recruitment). Items within the survey were not randomised. Adaptive questions were built into the survey (i.e., if a participant answers ‘yes’ to a question where specific details are required, further items or an open text box will be available for these details). All items required a response for completeness (noting that items where survey branching and logic applied, also required a response to these additional items). The survey could not be submitted if answers were missing, though the option of ‘not applicable’ was available on some items if the participant preferred not to respond. Participants were able to review their responses and adjust if they wished prior to submitting the survey.

The survey was pilot tested during the project planning phase with a sample of the targeted population. The survey was bespoke and developed for the purposes of this research, and was expected to take 20 mins with a total of 149 items across the survey, including all branching logic. The survey was developed recognising prior work [in^7^, ^22^]. The survey was tested by: (i) a medical professional who has experience of treating concussion patients; (ii) an allied health professional who has experience of treating concussion patients; (iii) an allied health professional who has not had experience of treating concussion patients; and (iv) a health professional that works with Aboriginal and Torres Strait Islander people. Utility and technical functionality of the electronic questionnaire were also pilot tested prior to its wider dissemination. Consumer feedback from pilot testing was embedded into the final version of the survey (available upon request). The final survey included a maximum of 10 survey items per page (without branching logic) and was completed over no more than 15 screens. Survey items were concise to increase completion rate.

Convenience sampling was used for data collection. The survey was disseminated via email and social media, which allowed for snowballing. Social media platforms included Facebook, X and LinkedIn, and targeted contact by email with specific groups, such as hospitals, Aboriginal medical services, local councils, aged care facilities, sporting groups, domestic and family violence associations, professional associations, early learning services, and schools. With this approach, organisations/groups were requested to share the survey invitation internally within their organisations or via their marketing communications (e.g. newsletters), individuals were not targeted. Survey representation was sought across all Australian States and Territories and included both metropolitan and rural/regional areas. Surveys were also accessed via a QR code. An incentive (chance to win a single Apple iPad via a draw) was offered to participants for their participation in the study with participants able to opt in at the end of the survey.

### Data analysis

Quantitative methods of analysis were primarily adopted using non-parametric approaches, and this was supplemented by content analysis for the free text responses. Binary and categorical data were presented as a number and a percentage, and continuous data were presented as a mean and standard deviation (SD) for descriptive data. Researchers (SW, LL) adopted a qualitative content analysis approach, informed by a naturalist paradigm [23]. Where appropriate, open text responses were independently coded by two researchers (SW, LL). Open text responses were coded to common categories with categories derived inductively from the data. Researchers (SW, LL) compared codes across open text items refining the category definitions, discrepancies were discussed and resolved by consensus. Final categories were summarised and reported as a frequency count and percentage to convey prevalence. To enhance trustworthiness, we used independent double-coding with consensus resolution and maintained an audit trail of coding decisions to support credibility and transparency. For any open text response where there was a typographical error, an unknown abbreviation, or a response that did not align with the question, these data were excluded from the content analysis. Health professionals’ location in which they deliver care was manually coded by a researcher (SW) using the Modified Monash Model [24]. Modified Monash Model defines whether a location is metropolitan (MM1), regional (MM2), a large rural town (MM3), a medium rural town (MM4), a small rural town (MM5), a remote community (MM6), or a very remote community (MM7), and is informed by the Australian Bureau of Statistics’ national census conducted every 5 years [24].

Several non-parametric correlation analyses (Chi-squared test) were completed to report the correlation between the survey participant’ characteristics and associated responses. Correlations between survey participant characteristics and survey responses included: (i) survey participant characteristics: patient population treated (i.e. paediatrics or adults; Aboriginal and Torres Strait Islander peoples), professional background (i.e. medical, nursing or allied health), working location (metropolitan (i.e. MM1) or regional/remote (i.e. ≥MM2)); (ii) continuous variables: health professional confidence to make a concussion diagnosis, health professional confidence to manage concussion, health professional confidence to differentiate between a concussion and a more severe head injury; and (iii) categorical variables: health professional reliance on published guidelines. In addition, correlations between different survey responses included the number of patients seen by health professionals for diagnosis, treatment and management, and the correct identification of signs and symptoms of concussion, confidence levels (confidence to differentiate between a concussion and a more severe head injury, and confidence to make a concussion diagnosis), and confidence to manage a concussion. The Pearson correlation coefficient was used to assess the linear relationship between continuous variables, and the Spearman correlation coefficient was used to assess the relationship between categorical variables. A Pearson correlation was completed to assess the relationship between demographic data (e.g. professional background and experience) and confidence of health professionals. A Pearson correlation was completed to assess the relationship between: (i) professional background; (ii) clinical experience; (iii) patient population (paediatrics, adults, older adults); (iv) number of people assessed for a suspected concussion by the health professional; (v) number of concussion diagnoses made by the health professional; (vi) number of people treated for concussion by a health professional; (vii) working location (metropolitan/regional); and patient populations including Aboriginal and Torres Strait Islander peoples versus those that didn’t; and confidence of health professional to (i) make a diagnosis of concussion; (ii) manage a concussed person within the scope of their professional background; (iii) differentiate between concussion and a more severe head injury. A Spearman correlation was completed to assess the relationship between: (i) professional background; (ii) clinical experience; (iii) patient population (paediatrics, adults, older adults); (iv) number of people assessed for a suspected concussion by the health professional; (v) number of concussion diagnoses made by the health professional; (vi) number of people treated for concussion by the health professional; (vii) working location (metropolitan/regional); and patient populations including Aboriginal and Torres Strait Islander peoples versus those that didn’t; and health professional (a) reliance of concussion guidelines; and (b) concussion symptoms.

Due to the anonymous nature of the survey, once survey responses were submitted, they were unable to be withdrawn. Incomplete questionnaires were not included in the analysis. View rates of the survey and recruitment rates were not reported, as unique visitor identifiers were not collected. Cookies were not used (outside of what is standard in accordance to REDCap practices) to identify individual participants. Log file analysis (e.g., reviewing for atypical time stamps) and IP address checking did not occur. The completion rate was reported by dividing the number of people who finished the survey (numerator) by the number of people who agreed to participate (i.e., complete and incomplete surveys; denominator).

## Results

### Participants

Of the 132 participants who completed the survey (*n* = 97 incomplete surveys; completion rate 58%), six participants were excluded: four participants had not worked professionally in the last year, one participant was not a health professional, and one participant worked overseas. Of the included 126 health professionals who participated in this study, 39 (31%) were physiotherapists, 23 (18%) were occupational therapists, 17 (14%) were nurses and 13 (10%) were medical professionals; most of which had over 10 years of experience (Table 1). Most participants were aged between 30 and 39 years (*n* = 38/126, 30%) and 98 (78%) were female. English was the most reported language spoken by health professionals in their professional role with patients (*n* = 124/126, 98%), followed by Italian (*n* = 27/126, 21%), and Mandarin (*n* = 26/126, 21%) (Supplementary Table 1). While the a priori target sample size was not achieved due to slower than expected recruitment, the sample met the a priori sample representation targets for gender, health professional groups and rurality (Table 1).

**Table 1:** Participant demographics

	Health professionals (*n* = 126)
**Gender** (*n*, %)	
Male	27 (22%)
Female	98 (78%)
**Age** (n, %)	
18 to 29 years	16 (13%)
30 to 39 years	38 (30%)
40 to 49 years	32 (25%)
50 to 59 years	33 (26%)
60 to 69 years	6 (5%)
70 to 79 years	1 (1%)
**Professional background** (n, %)	
Aboriginal Health Worker	1 (1%)
Aboriginal Family Support Worker	1 (1%)
Aboriginal Family Support Worker	1 (1%)
Clinical Psychologist	4 (3%)
Emergency Medicine Registrar/Specialist	3 (2%)
General Practitioner	3 (2%)
Geriatrician	4 (3%)
Neuropsychologist	2 (2%)
Nurse	17 (14%)
Occupational Therapist	23 (18%)
Other relevant community experience^±^	5 (4%)
Other^†^	20 (16%)
Paediatrician	1 (1%)
Physiotherapist (general)	39 (31%)
Physiotherapist (speciality)	
Cardiorespiratory	5 (4%)
Gerontology	3 (2%)
Musculoskeletal	18 (14%)
Neurology	9 (7%)
Paediatrics	2 (2%)
Pain	6 (5%)
Sports and Exercise	9 (7%)
Women’s, Men’s and Pelvic Health	1 (1%)
Vestibular	5 (4%)
N/A	2 (2%)
Other^†^	5 (4%)
Psychiatrist/Neuropsychiatrist	1 (1%)
Speech Pathologist	2 (2%)
Sports Medicine Specialist	1 (1%)
Social Worker	5 (4%)
**Years of clinical experience** (n, %)	
Less than one year	5 (4%)
Between 1–2 years	7 (6%)
Between 3–4 years	5 (4%)
Between 5–9 years	21 (17%)
Between 10–14 years	25 (20%)
Between 15–19 years	12 (9%)
Between 20–24 years	20 (16%)
More than 25 years	31 (24%)
**Currently employed** (n, %)	120 (95%)
**Paid hours worked** (mean (SD), range)	32.94 (10.08), 3–76
**Sector of employment** (n, %)	
Public sector	45 (36%)
Private sector	77 (66%)
Other^†^	12 (10%)
**Patient population health professional treats** (n, %)	
Paediatrics (0–17 years)	29 (23%)
Adults (18–64 years)	84 (67%)
Older adults (≥ 65 years)	68 (54%)
Birth to end of life	31 (25%)
**Patient population identifies as Aboriginal or Torres Strait people** (n, %)	
Aboriginal	30 (24%)
Torres Strait Islander	0 (0%)
Aboriginal or Torres Strait Islander	67 (53%)
**Health professional directs patients towards resources and services for Aboriginal or Torres Strait Islander people** (n, %)^µ^	
Yes	28 (42%)
No	19 (28%)
Not available where I work	15 (22%)
Not applicable	5 (8%)
**Location by state** (n, %)^¶^	
New South Wales	28 (22%)
Northern Territory	3 (3%)
Queensland	16 (13%)
South Australia	6 (5%)
Tasmania	14 (11%)
Victoria	48 (38%)
Western Australia	9 (7%)
Works across multiple states	1 (1%)
**Rurality** (n, %)^¶^	
Metropolitan (MM1)	72 (57%)
Regional (MM2)	22 (18%)
Large rural town (MM3)	6 (5%)
Medium rural town (MM4)	5 (4%)
Small rural town (MM5)	11 (9%)
Remote community (MM6)	1 (1%)
Very remote community (MM7)	3 (2%)
Works across a number of regions classified differently on MMS	5 (4%)

^±^Includes Aboriginal and Torres Strait Islander health workers whereby cultural, community, or lived experience contributes to their recognised role in supporting community health; ^µ^only includes participant responses of those that work with Aboriginal and/or Torres Strait Islander Peoples (n = 67); ^¶^missing data (n = 1); ^†^ ’Other’ responses reported in Supplementary Table 2

### Mechanisms of concussion injuries and symptom identification

**Fig. 1:**
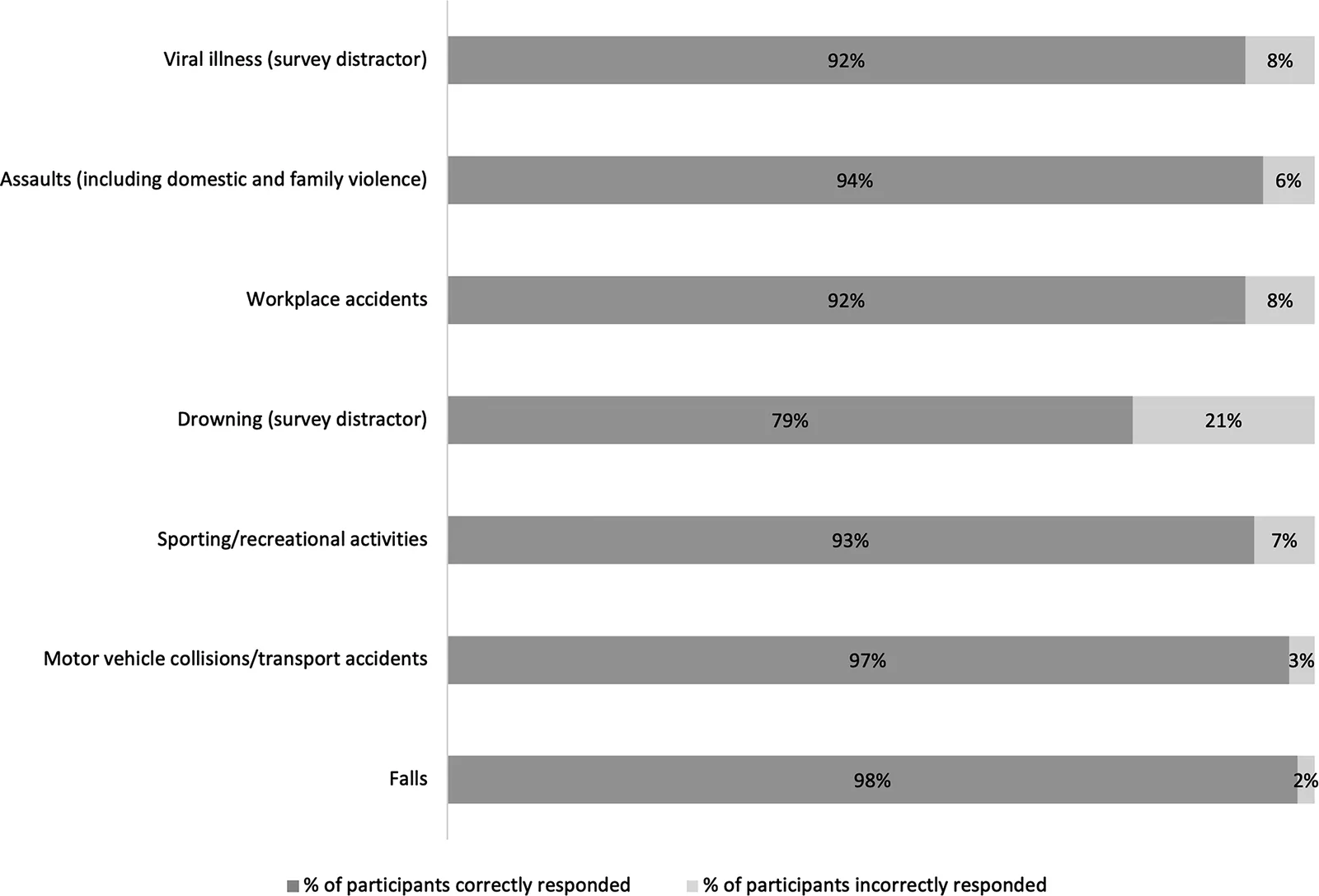
Identification of the common causes of concussion

**Fig. 2:**
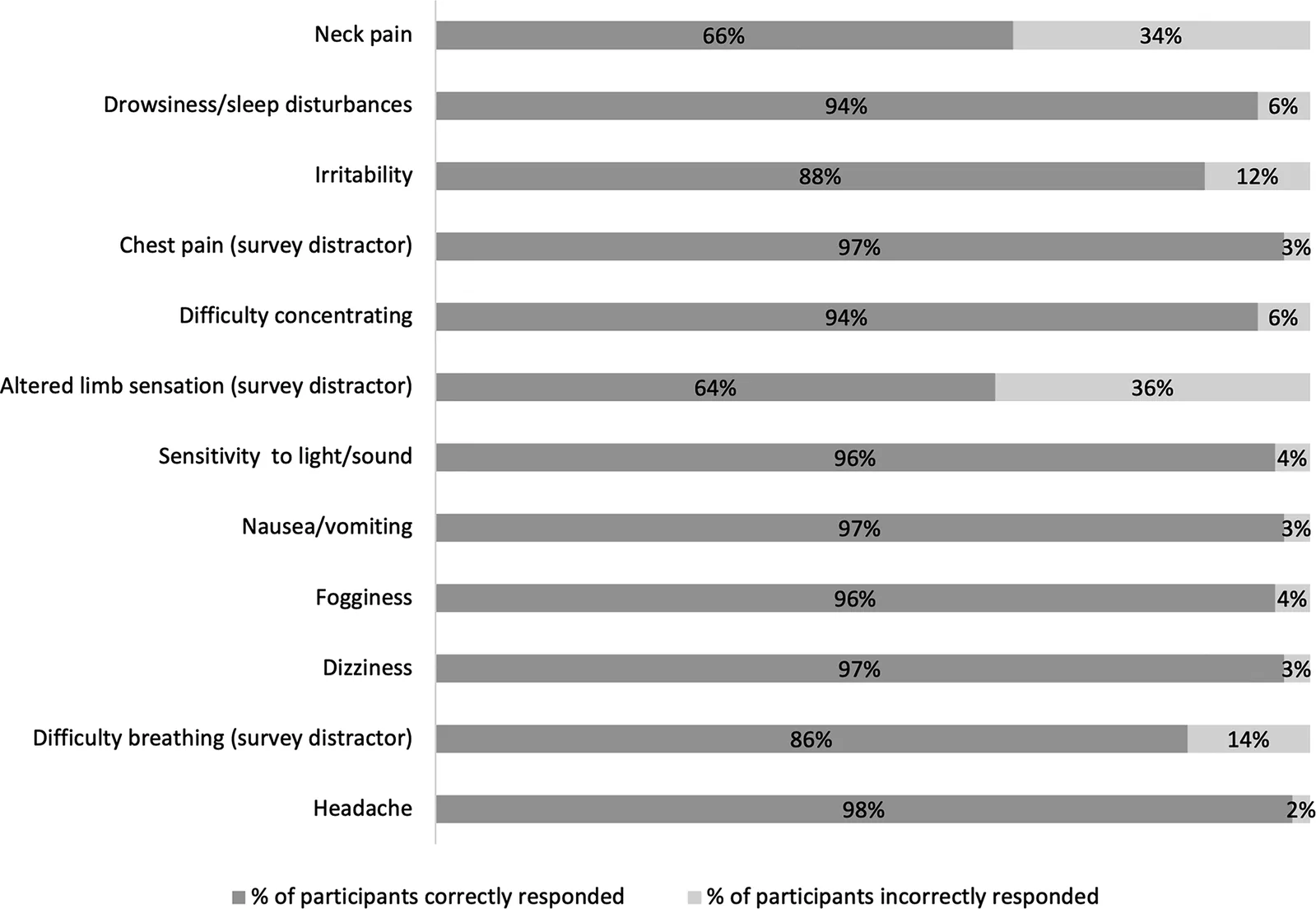
Correct identification of concussion symptoms

Of the 126 participants, 85 (68%) reported all the common causes of concussion from a predetermined list (Fig. 1). Thirty participants (24%) identified all the symptoms indicative of concussion (Fig. 2). However, when symptoms were examined individually rather than as a group, correct identification was higher. In the free-text responses, overlap was evident between the symptoms reported as free text and the pre-determined list (e.g., visual disturbances, mood problems) (Supplementary Table 2).

**Fig. 3:**
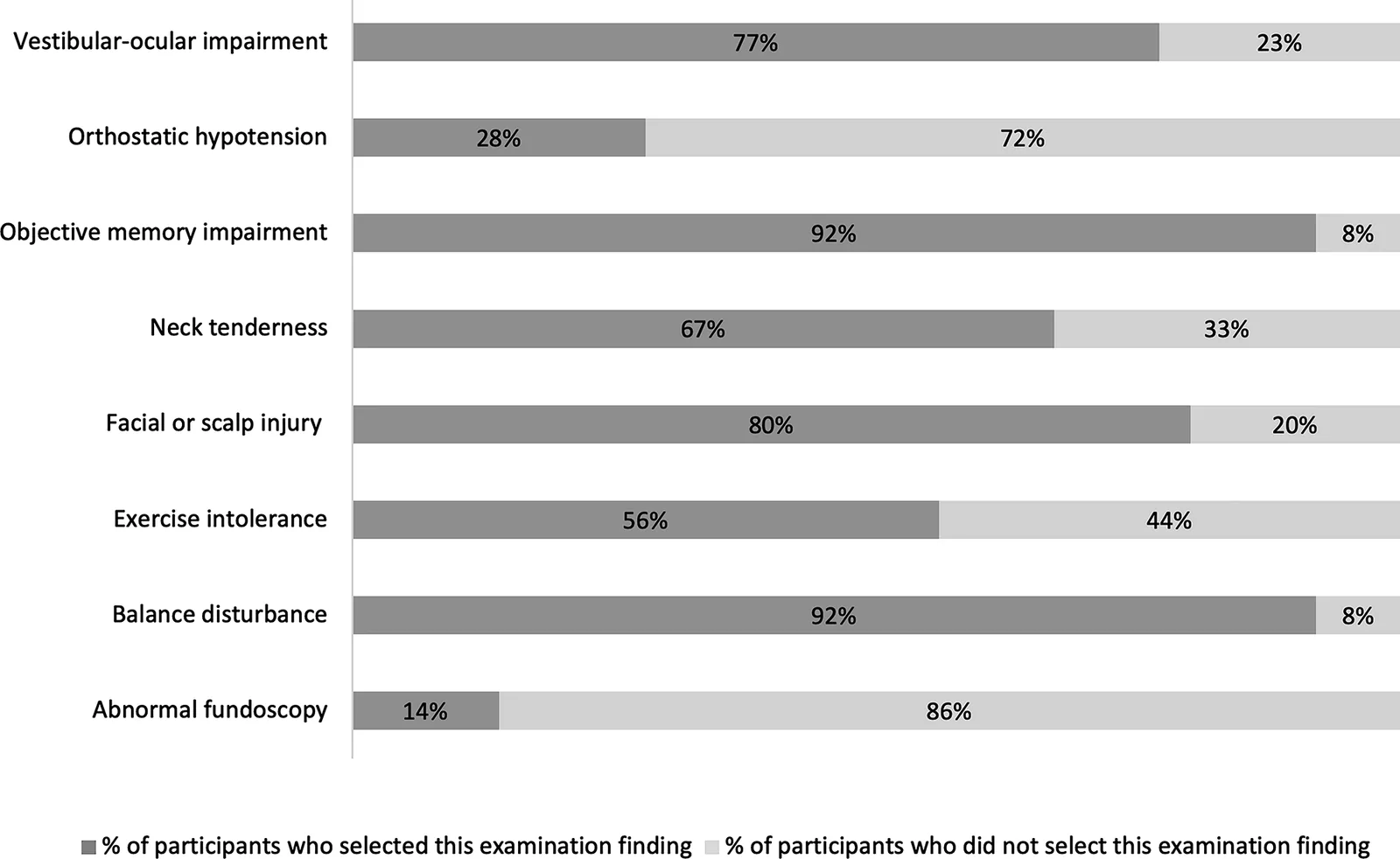
Examination findings (if applicable) used by health professionals that may indicate a patient has sustained a concussion (*n* = 92)

There were 34 participants who indicated they would not consider examination findings to be within their current scope of practice, and for the other 92 participants, there was variability in the selection of the examination findings that may be suggestive of concussion (Fig. 3). In the free-text responses, there was a variance in examination findings reported (Supplementary Table 2).

### Published guidelines and information that guides concussion management practices for health professionals

Over half of the health professionals indicated that they would ‘frequently’ (*n* = 65/126, 52%) rely on published clinical guidelines (Table 2). Of the small number of participants (*n* = 4/126, 3%) that reported they ‘never’ rely on published guidelines, two reported that it was because they were unaware that guidelines exist (Table 2). From a pre-determined list, 54 (43%) participants identified that they were aware of the Australian Institute of Sport (AIS) Position Statements, and 30 (24%) were aware of the ‘Living Concussion Guidelines Guideline for Concussion & Prolonged Symptoms for Adults 18 years of Age or Older’ (Table 2).

**Table 2:** Knowledge of guidelines and information to support concussion management

	Health professionals (*n* = 126)
**Frequency health professionals rely on published guidelines** (*n*, %)	
Never	4 (3%)
Rarely	8 (6%)
Sometimes	34 (27%)
Frequently	65 (52%)
Always	15 (12%)
**Reported reasons of why published guidelines not referred to** (n, %)	
Health professional(s) were not aware that guidelines exist	2 (2%)
Other^†^	2 (2%)
**Awareness of specific published guidelines for concussion** (n, %)	
HealthPathways	19 (15%)
Australian Institute of Sport (AIS) Position Statements	54 (43%)
Concussion in Brain Health Position Statement 2024	40 (74%)
Concussion and Brain Health Position Statement 2023	12 (22%)
Concussion in Sport Australia Position Statement 2019	5 (9%)
Concussion in Sport Position Statement 2016	1(2%)
Living Concussion Guidelines Guideline for Concussion & Prolonged Symptoms for Adults 18 years of Age or Older	30 (24%)
Australian and New Zealand Guideline for Mild to Moderate Head Injuries in Children	26 (21%)
PedsConcussion Living Guideline for Pediatric Concussion Care	8 (6%)
CDC Guideline on the Diagnosis and Management of mTBI among children	7 (6%)
Other^†^	13 (10%)
**Sources of information for concussion management**	
Google	51 (41%)
HealthPathways	35 (28%)
Websites^†^	42 (33%)
Colleagues (same profession)	71 (56%)
Colleagues (different profession)	55 (44%)
Other^†^	23 (18%)

^†^ Description of the websites referred to for concussion management and ’Other’ responses are reported in Supplementary Table 2

### Health professionals’ knowledge and experience of managing concussion

**Fig. 4:**
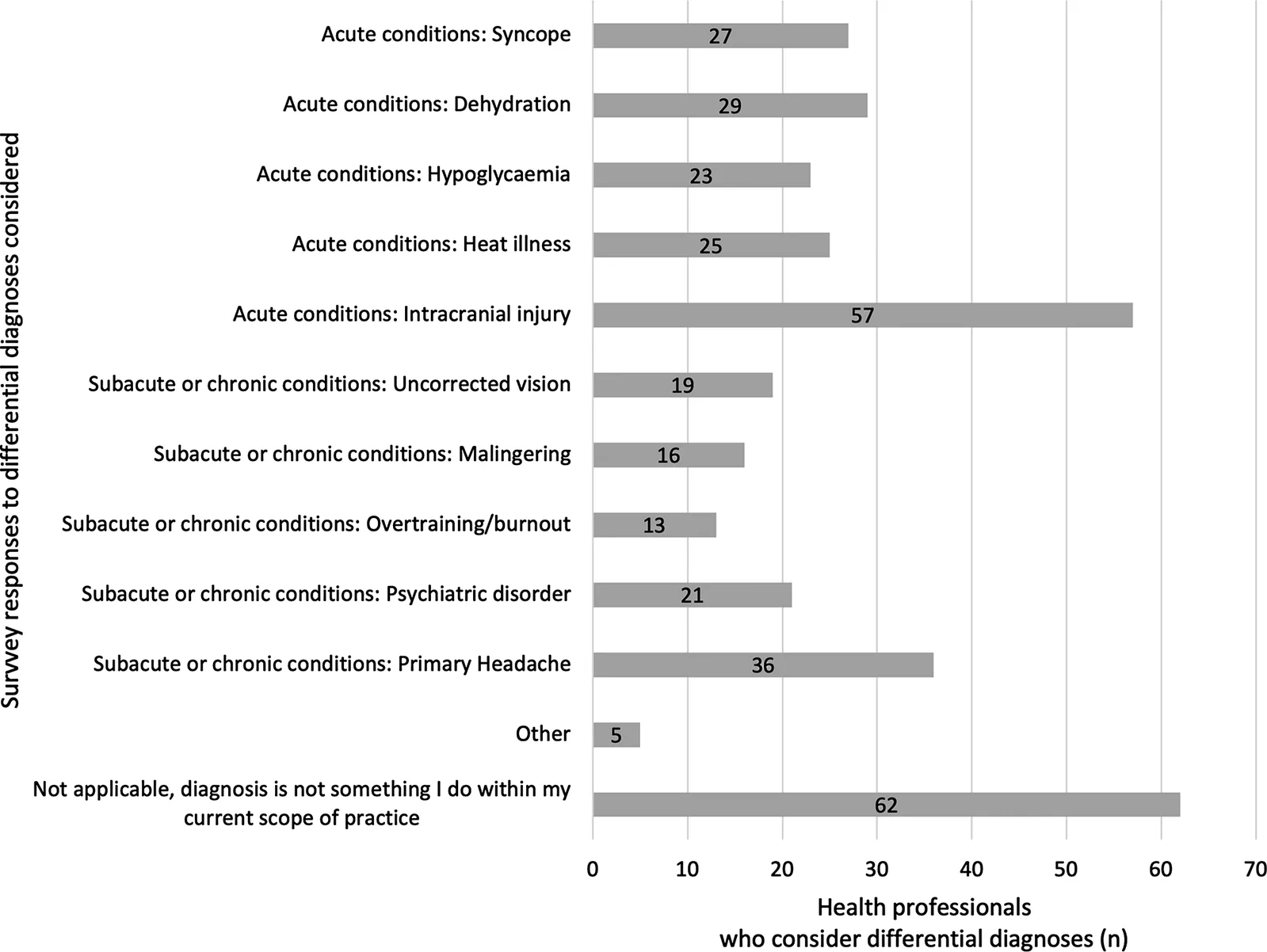
Differential diagnoses

**Fig. 5:**
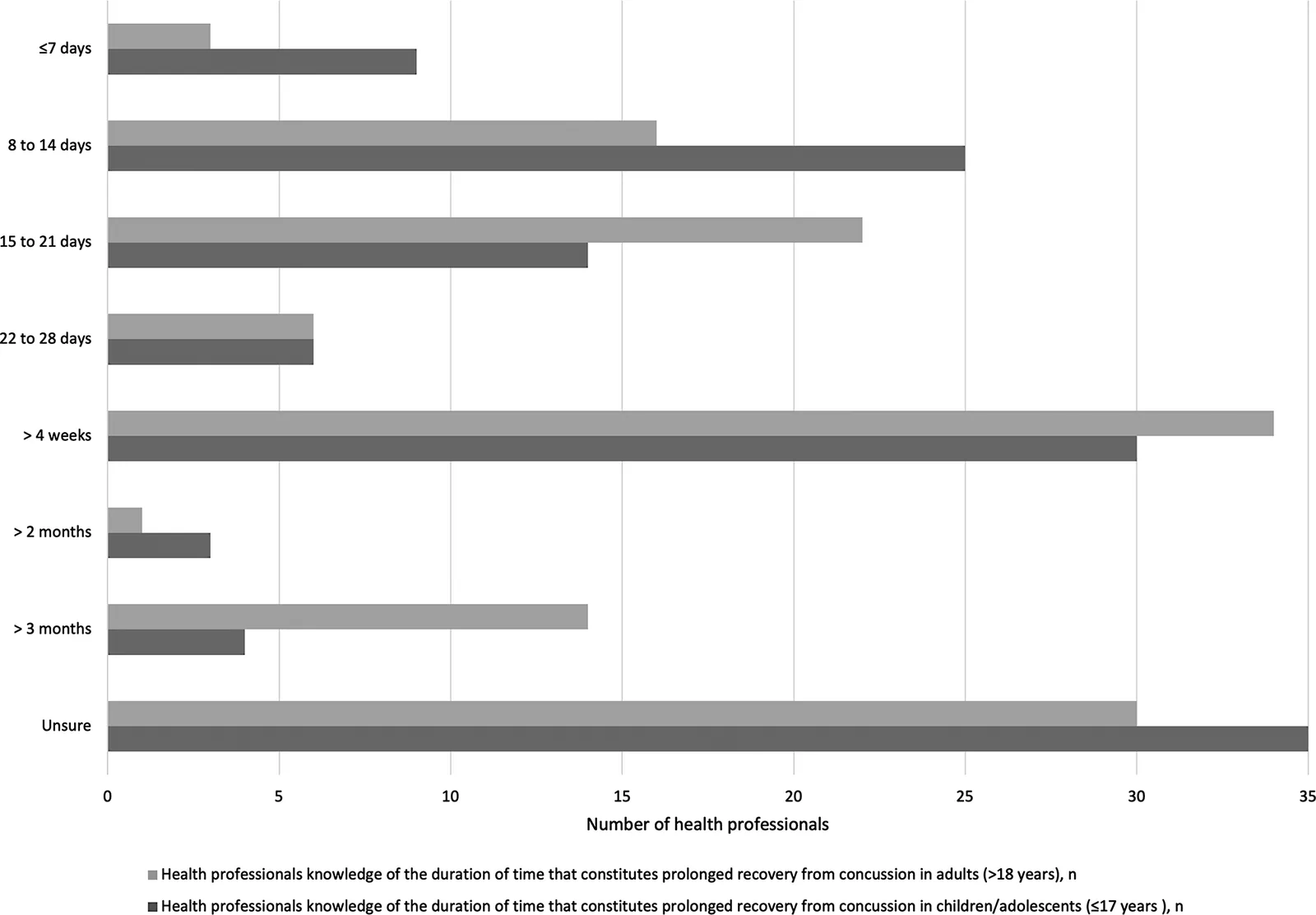
Health professionals’ perception of the duration that constitutes prolonged recovery in children and adults

**Fig. 6:**
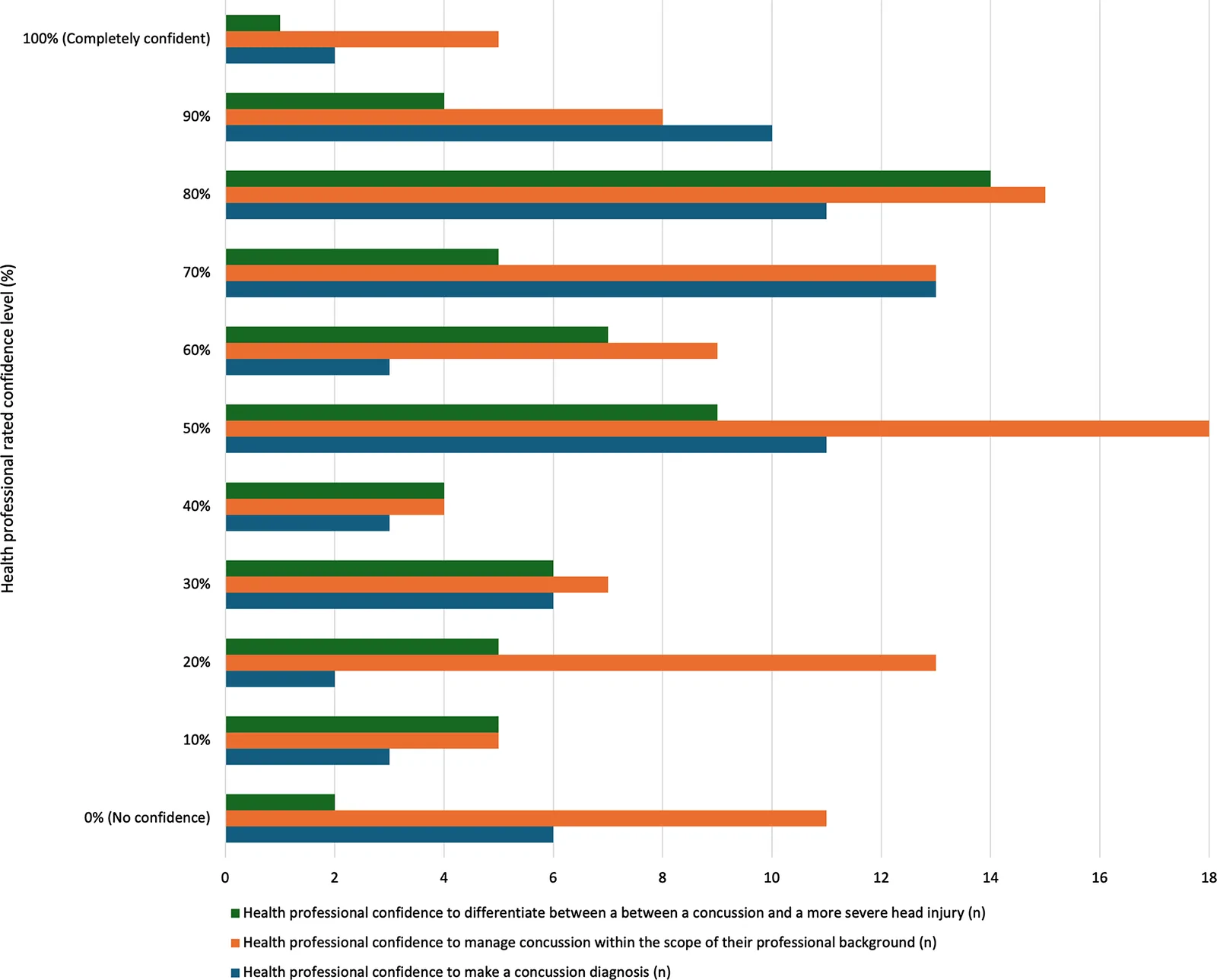
Health professional confidence levels with differentiating concussion and other head injuries, managing and diagnosing concussion

Seventy health professionals (56%) reported they were involved with assessing people with a suspected concussion, of which 53 (42%) reported that they had diagnosed concussion within the last 3 months (noting that only 11 participants reported they were a medical professional) (Table 3). More than 50% of participants (*n* = 66/126, 52%) reported they treated concussion within an average 3-month period (Table 3). Health professionals reported frequent referral of patients to other medical professionals (GP and Emergency Medicine) (Table 3). There was variation across reporting about the frequency in which health professionals would review or treat a patient with concussion, with majority reporting either weekly (*n* = 25/66, 38%), or monthly (*n* = 10/66, 15%) (Table 4). In terms of concussion being a secondary diagnosis, where applicable, it was reported to ‘rarely’ occur in clinical practice (*n* = 54/126, 43%) (Supplementary Table 3). Only a small cohort of participants (*n* = 39/126, 31%) utilised diagnostic imaging in their practice. For those that did, a combination of Magnetic Resonance Imaging (MRI) (*n* = 10/20, 50%) and Computed Tomography Scan (CT) (*n* = 14/20, 70%) was reported to be frequently requested (noting that diagnostic imaging is often used to exclude more severe head injury) (Table 5). In addition, other diagnostic assessments were used: most frequently reported was clinical interview (*n* = 59/73, 81%), followed by the Sport Concussion Assessment Tool (SCAT; any version) (*n* = 22/73, 30%) (Table 5). In terms of ongoing concussion management, key assessments reported were clinical interview (*n* = 57/74, 77%), and cognitive assessments (*n* = 33/74, 45%) (Table 6). Within the free text responses, the most frequently reported cognitive assessment was the Westmead Post-Traumatic Amnesia Scale/Abbreviated Westmead Post-Traumatic Amnesia Scale (*n* = 7/28, 25%) (Supplementary Table 2). For health professionals who manage a person with persisting concussion symptoms (beyond four weeks following injury), most reported they would refer to a medical specialist (*n* = 47/75, 63%), or refer to a publicly funded health service for outpatient care (*n* = 36/75, 48%) (Table 6). Further, regarding medical specialists that health professionals would refer a person with persisting symptoms to, neurologists and general practitioners (GPs) were most frequently mentioned in the open text responses (Supplementary Table 2). Most participants indicated that they had access to written patient education on concussion, however, only about half of the participants indicated they would provide this information ‘most of the time’ or ‘all of the time’, with most relying on written information from the Department of Health (*n* = 38/58, 66%) (Table 7). Health professionals were mainly influenced by their ‘own clinical judgement’ (*n* = 60/79, 76%) in terms of the assessments used for diagnosis and/or ongoing management and monitoring of concussion, with a smaller number following recommendations provided by their place of employment (*n* = 34/79, 43%). Differential diagnoses were considered by health professionals who diagnose concussion, with intracranial injury (*n* = 57/64, 89%) being the most frequently reported differential diagnosis considered (Fig. 4). There were several other differential diagnoses reported as part of the open text responses, e.g., Benign Paroxysmal Positional Vertigo (BPPV; *n* = 2/13, 15%), Attention Deficit Hyperactivity Disorder **(**ADHD; *n* = 1/13, 8%) and autism (*n* = 1/13, 8%) (Supplementary Table 2). There were 78 (62%) health professionals that reported knowledge of persistent post-concussion symptoms, with > 4 weeks considered to be the most frequently reported duration of prolonged recovery in both children (*n* = 30/126, 24%) and adult (*n* = 34/126, 27%) populations, however several health professionals thought it would be earlier in both populations, or they were unsure (Fig. 5). Familiarity with Chronic Traumatic Encephalopathy (CTE) varied from ‘no knowledge’ (*n* = 37/126, 29%) through to ‘very familiar’ (*n* = 3/126, 2%) (Supplementary Table 4), with over half of health professionals indicating that CTE has been associated with repeated head injuries (*n* = 91/126, 72%). When asked about knowledge of CTE symptoms, over half of participants responded to each feature of CTE correctly (Supplementary Fig. 1). Confidence to diagnose (noting participants that indicated that concussion diagnosis was outside of their scope of practice were not included), manage and differentiate concussion from other more severe head injuries ranged from 0% (no confidence) to 100% (completely confident), with more participants responding > 50%, indicating a higher level of confidence (Fig. 6).

**Table 3:** Participants current practices in patient care for concussion (assessment, diagnosis, treatment and ongoing management) (*n* = 126)

	Management of a suspected concussion	Diagnosis of concussion	Treatment and ongoing management of concussion		
	*Number of people assessed for suspected concussion within a 3-month period by participants,* (*n*, %)	*Number of people a health professional has diagnosed with concussion within a 3-month period,* (*n*, %)	*Number of patients discharged from hospital after initial management of concussion to health professional for ongoing management*	*Number of people a health professional has treated after diagnosis within a 3-month period,* (*n*, %)	*Number of people a health professional has referred on for ongoing management by another profession within a 3-month period for concussion, (n, %)*
0 people	56 (44%)	73 (58%)	33 (50%)	60 (48%)	63 (50%)
≤ 5 people	51 (41%)	40 (32%)	25 (38%)	50 (40%)	49 (39%)
6–10 people	9 (7%)	8 (6%)	4 (6%)	10 (8%)	12 (9%)
11–30 people	10 (8%)	5 (4%)	4 (6%)	5 (4%)	2 (2%)
≥ 51 people	-	-	-	1 (1%)	-
	*If 0 people assessed*,* referrals made to another health professional*^¶^	*If 0 people diagnosed with concussion*,* referrals made to another health professional*^¶^		*If 0 people treated with concussion*,* referrals made to another health professional*^¶^	
Yes	7 (12%)	17 (23%)		18 (30%)	
No	18 (32%)	12 (16%)		5 (8%)	
Not applicable	21 (55%)	44 (60%)		37 (62%)	
	*Health profession referred to for assessment of suspected concussion*^¶^ (*n* = 7)	*Health profession referred to for diagnosis* ^¶^ (*n* = 17)		*Health profession referred to for treatment* ^¶^ (*n* = 18)	*Other health professionals engaged in care* (*n* = 63)
Physiotherapist (general)	1 (14%)	3 (6%)		3 (17%)	22 (35%)
Physiotherapist (speciality)					
Musculoskeletal	-	-		-	7 (32%)
Neurology	1 (14%)	1 (6%)		1 (6%)	10 (45%)
Paediatrics	-	-		-	2 (9%)
Sports and Exercise	-	-		-	4 (18%)
Vestibular	-	2 (12%)		2 (11%)	12 (55%)
Occupational Therapist	1 (14%)	-		3 (17%)	24 (38%)
Speech Pathologist	-	-		-	7 (11%)
Optometrist	-	-		-	8 (13%)
Clinical Psychologist	-	-		-	12 (19%)
Neuropsychologist	1 (14%)	1 (6%)		3 (17%)	16 (25%)
Exercise Physiologist	-	-		-	6 (9%)
Dietitian	-	-		-	2 (3%)
Sleep Specialist	-	-		-	2 (3%)
Nurse	-	-		-	5 (8%)
Social Worker	-	-		-	7 (11%)
Medical professionals					
General Practitioner	4 (57%)	8 (47%)		7 (39%)	31 (49%)
Emergency Medicine Registrar/Specialist	5 (71%)	11 (65%)		6 (33%)	8 (13%)
Neurologist	-	2 (12%)		2 (11%)	16 (25%)
Psychiatrist/Neuropsychiatrist	-	1 (6%)		2 (11%)	3 (5%)
Sports Medicine Specialist	1 (14%)	2 (12%)		-	13 (21%)
Pain Management Specialist	-	-		-	3 (5%)
Rehabilitation Specialist	1 (14%)	3 (18%)		-	18 (29%)
Paediatrician	-	2 (12%)		-	3 (5%)
Geriatrician	-	-		-	5 (8%)
Other^†^	-	3 (18%)		1 (6%)	5 (8%)

^¶^ Participant responses only for those that report 0 people assessed, diagnosed and treated for concussion within a 3-month period; ^†^ ’Other’ responses reported in Supplementary Table 2

**Table 4:** Health professionals frequency of review or treatment of a patient with concussion post-diagnosis

Frequency health professional would review or treat a patient with a concussion, *n* (%)	Health professionals^¶^ (*n* = 66)
Daily	8 (12%)
Weekly	25 (38%)
Monthly	10 (15%)
Bi-monthly	7 (11%)
Quarterly	4 (6%)
Six-monthly	1 (2%)
Yearly	3 (5%)
Other^†^	8 (12%)

^†^ ’Other’ responses reported in Supplementary Table 2; ^¶^Health professionals, including medical professionals

**Table 5:** Health professionals’ knowledge regarding diagnostic practices related to concussion management

	Health professionals (*n* = 126)^¶^
**Referral made for diagnostic imaging for concussion** (*n*, %)	
Yes	20 (16%)
No	19 (15%)
Not applicable, this is not something I do within current scope	87 (69%)
**Types of diagnostic imaging requested for concussion** (n, %)	
Magnetic Resonance Imaging (MRI)	10 (50%)
Computed Tomography Scan (CT)	14 (70%)
Other^†^	2 (10%)
**Diagnostic assessments used**^¶^ (n, %)	
Clinical interview	59 (81%)
Sport Concussion Assessment Tool (any version)	22 (30%)
SCAT6™	11 (50%)
SCAT5™	7 (32%)
SCAT2™	1 (4%)
SCAT™	3 (14%)
Child Sport Concussion Assessment Tool (any version)	8 (11%)
Child SCAT6™	7 (88%)
Child SCAT5™	1 (12%)
Sport Concussion Office Assessment Tool 6 (SCOAT6™)	11 (15%)
Child Sport Concussion Office Assessment Tool 6 (Child SCOAT6™)	6 (8%)
CDC Acute Concussion Evaluation (ACE)	5 (7%)
Concussion-specific standardised questionnaires	24 (33%)
Symptom specific standardised questionnaires	13 (18%)
Cognitive assessments	21 (29%)
Battery of assessment(s) as determined by the health professionals’ local health service policies and procedures^†^	8 (11%)
Other^†^	5 (7%)

^†^ ’Other’ and ‘Battery of assessment(s) as determined by the health professionals’ local health service’ responses reported in Supplementary Table 2

**Table 6:** Health professionals’ knowledge of ongoing concussion management

	Health professionals (*n* = 126)
**Assessments used by health professionals for ongoing management and monitoring of concussion,** ^¶^ *n* (%)	
Clinical Interview	57 (77%)
Post-Concussion Symptom Scale (PCSS) or Rivermead Post Concussion Symptoms Questionnaire (RPQ)	23 (31%)
Computerised Neurocognitive testing^†^	5 (7%)
Cognitive assessments^†^	33 (45%)
Return-to-Sport/Learn/Activity Protocol	18 (24%)
HeadCheck App or other	3 (4%)
Other^†^	9 (12%)
**Management of with concussion where symptoms persisted beyond 4 weeks**, ^Π^ n (%)	
No further management is needed, symptoms will resolve eventually	0 (0%)
Referral to a medical specialist^†^	47 (63%)
Referral to publicly funded health service for outpatient care	36 (48%)
Refer to a privately owned Concussion Clinic	21 (28%)
Close observation	10 (13%)
Weekly	5 (50%)
Fortnightly	2 (20%)
Other frequency	3 (30%)
Other management approach^†^	8 (11%)

^¶^ Excludes n = 47 (where responses were ‘Not applicable, this is not something I do within my current scope of practice’); ^Π^Excludes n = 51 (where responses were ‘Not applicable, this is not something I do within my current scope of practice’); ^†^ ’Other’ responses, computerised neurocognitive and cognitive assessments used, and descriptions of medical specialists have been reported in Supplementary Table 2

**Table 7:** Access to patient concussion resources

	Health professionals (*n*, %)
**Access to written patient education on concussion (electronic or hardcopy versions available),** *n* (%)	
Yes	58 (46%)
No	45 (36%)
Not applicable	23 (18%)
**Frequency of written information to patients on concussion** ^µ^, n (%)	
None of the time	11 (19%)
Some of the time	12 (21%)
About half of the time	2 (3%)
Most of the time	17 (29%)
All of the time	16 (28%)
**Type of information provided** ^µ^, n (%)	
Written information from the Department of Health	38 (66%)
Refer patients to websites to review in their own time^†^	14 (24%)
Other^†^	13 (22%)

^µ^ Reporting only on responses where health professionals have indicated that they have access to written information (n = 58); ^†^ ’Other’ responses and websites described as part of open text responses are reported in Supplementary Table 2

### Participant factors influencing concussion knowledge

The only negative correlation identified was between clinical psychologist professional background and confidence to manage a concussed person with the scope of their professional background, r(125)= -0.19, *p* < 0.05. We found that as health professionals’ number of patients assessed for a suspected concussion, number of concussion diagnoses made, and the number of people treated for concussion their confidence increased (Table 8). No other correlations were identified (e.g. between working location and confidence of the health professional).

**Table 8:** Participant factors and confidence correlations

	Number of people a health professional assessed for suspected concussion within a 3-month period (*n* = 126)	Number of people a health professional diagnosed with concussion within a 3-month period (*n* = 126)	Number of people a health professional treated after diagnosis of concussion within a 3-month period (*n* = 126)
Confidence of health professional to make a diagnosis of concussion	0.61* ^µ^	0.64* ^µ^	0.39* ^µ^
Confidence of health professional to manage a concussed person within the scope of their professional background	0.22*	0.21*	0.16
Confidence of health professional to differentiate between a concussion and a more severe head injury	0.19*	0.25*	0.22*

*Correlation is significant at the 0.05 level (two-tailed); ^µ^only including participants who self-reported diagnosing concussion within their scope of practice

No correlations were identified, except for a significant negative relationship between speech pathologists and the frequency that published guidelines were relied upon, rs(126) = -0.18, *p* < 0.05, and that health professionals that work with Aboriginal and Torres Strait Islander peoples more frequently relied upon published guidelines, rs(126) = 0.27, *p* < 0.05.

## Discussion

This study provides insights into the varied knowledge that health professionals have in diagnosing and managing concussion in Australia. Health professionals demonstrated more accurate identification of concussion causes when compared to the complete identification of all concussion symptoms. For those participants that did consider concussion specific examination findings, there was variance in the reported responses, indicating varied practice methods. The frequent reference to published guidelines was seen to be a strength of the survey findings, however, there was variability in the guidelines that were referred to, which may be contextual to the timing of the survey data collection period, i.e. the ANZ Concussion Guidelines were released part-way through data collection. Over half of the participants of this survey reported they were involved in assessing people with a suspected concussion, and/or treated a person with a concussion as part of their current practice over the last 3-month period. Health professionals most frequently referred patients to general practitioners (GPs) in the acute phase of the injury and when the person’s symptoms persisted beyond four weeks, indicating the important role GPs play in the management of concussion. Health professionals demonstrated variability in their confidence levels to diagnose, manage and differentiate concussion from other more severe head injuries. Positive correlations were determined between participants’ self-reported confidence and the number of people seen with a suspected concussion, the number of diagnoses made or the number of people treated with concussion, and the frequency that health professionals referred to guidelines when considering if they treated Aboriginal and Torres Strait Islander People. These findings are consistent with national and international research demonstrating variability in concussion knowledge, confidence and practice among health professionals. Despite only three respondents being GPs in our study, prior research has shown that primary care doctors frequently manage concussion but report variable knowledge and inconsistent uptake of guidelines, further highlighting ‘GPs’ pivotal role’ in assessment, education and coordinated referral pathways [7, 8, 25].

The ANZ Concussion Guidelines emphasise the importance of patient education [11], however, this study found that the provision of concussion-related information to people following a concussion injury was not consistently provided by health professionals. Future research is warranted to explore the barriers in providing concussion-related patient education resources, including the presence of any age-specific limitations. It has previously been shown that when health professionals provided online educational resources (e.g., websites) for families of adults with moderate to severe brain injury, it was important to consider readability and reliability of the sources provided [26].

A proportion of the health professionals (38%) included in the study were unaware of persistent post-concussion symptoms (symptoms lasting for longer than four weeks [27]), indicating that patients may not receive adequate treatment to address their needs and therefore be at risk of poorer outcomes. While the inclusion of Persistent Post-Concussion Symptoms in the ANZ Guidelines is a promising step forward [11], this study reinforces the importance of concussion education for health professionals so that they recognise the frequency and timeline for persisting symptoms post-concussion, and the variable recovery trajectory. Another area that is emphasised in the ANZ Concussion Guidelines is the importance of considering differential diagnoses. Those health professionals where it was applicable as part of their scope of practice reported considering some differential diagnoses. This indicates that education, including knowledge translation of the guidelines, should target current knowledge gaps of health professionals, such as the identification and management of persistent post-concussion symptoms, timeframes for concussion recovery, possible differential diagnoses and associated clinical pathways to appropriately support post-concussion recovery. Specificity around education considering different health professionals, recognising that this survey predominately included physiotherapist and occupational therapists should be considered within this education framework (noting they do not diagnose concussion, however, are often involved in the care of a person prior to concussion diagnosis). Furthermore, education could be tailored and targeting to those health professionals that may not as commonly see people with concussion to improve confidence, recognising the positive correlation of confidence with the number of people seen in this study.

Despite maximal efforts by the research team to bolster recruitment for the study, it was not possible to achieve the target sample size. However, the sample did have representation across Australia from allied health, nursing and medical professionals. However, a limitation of the study may be the low number of GP and ED physicians included, and the low numbers of some groups for the correlation analyses. The a priori targets set for sampling considered representativeness of the health profession Australia wide, however, the male health professionals may have been underrepresented. While the survey was designed to be comprehensive, it is recognised that there may have not been the inclusion of all paediatric assessment scales as part of the response options for example, however, ‘other’ open text responses were available should the participant wish to provide details of any assessments not listed. Even though the ANZ Concussion Guidelines were ‘soft’ launched during the data collection period, only two health professionals referred to this guideline, emphasising the need for increasing awareness of this resource.

## Conclusions

Although many health professionals were familiar with mechanisms of concussion injuries, there is still varied understanding of concussion symptoms and knowledge (including differential diagnoses), its management and recovery expectations. This knowledge gap forms a framework for targeted education for health professionals who are involved in the treatment of patients with a suspected or actual concussion. Such education could complement the recently released ANZ Concussion Guidelines, with the potential to enhance translation of the guidelines into the clinical practice of Australian health professionals.

## Supplementary Information

Below is the link to the electronic supplementary material.

Supplementary Material 1

## Data Availability

The datasets used and analysed during the current study are available from the corresponding author on reasonable request.

## Abbreviations

TBI: Traumatic brain injury
REDCap: Research Electronic Data capture
GPs: General Practitioners
MRI: Magnetic Resonance Imaging
CT: Computed Tomography Scan
BPPV: Benign Paroxysmal Positional Vertigo
ADHD: Attention Deficit Hyperactivity Disorder
