# Supplementary material for: Understanding Australian health professionals’ knowledge of concussion: a cross-sectional nationwide survey
Source: BMC Health Serv Res. 2026 Aug 13;26:1578. doi: 10.1186/s12913-026-15122-9 (PMC13757058; doi:10.1186/s12913-026-15122-9)
Supplement: Supplementary file 1 [file 12913_2026_15122_MOESM1_ESM.docx]

Supplementary / Additional Material

**Title**: Understanding Australian health professionals’ knowledge of concussion: A cross-sectional nationwide survey.

Table of Contents

[Appendix 1: Checklist for Reporting Results of Internet E-Surveys (CHERRIES)	2](#_Toc209780188)

[Supplementary Table 1: Languages spoken by patient populations treated	3](#_Toc209780189)

[Supplementary Table 2: Content analysis of open text survey responses	4](#_Toc209780190)

[Supplementary Table 3: Frequency of concussion being a secondary diagnosis in clinical practice	16](#_Toc209780191)

[Supplementary Table 4: Health professional’ knowledge of chronic traumatic encephalopathy (CTE)	16](#_Toc209780192)

[Supplementary Figure 1: Health professional knowledge of symptoms of CTE	17](#_Toc209780193)

## **Appendix 1: Checklist for Reporting Results of Internet E-Surveys (CHERRIES)**

| **Item Category** | **Checklist Item** | **Page Number** |
| --- | --- | --- |
| Design | Describe survey design | Page 6 |
| Institutional Review Board Approval and Informed consent | IRB approval | Page 5 |
|  | Informed consent | Page 5 |
|  | Data Protection | Page 5 to 6 |
| Development and pre-testing | Development and testing | Page 6 to 7 |
| Recruitment process and description of the sample having access to the questionnaire | Open survey versus closed survey | Page 6 |
|  | Contact mode | Page 7 |
|  | Advertising the survey | Page 7 |
| Survey administration | Web/E-mail | Page 7 |
|  | Context | Page 5 |
|  | Mandatory/voluntary | Page 5 |
|  | Incentives | Page 7 |
|  | Time/Date | Page 6 |
|  | Randomisation of items or questionnaires | N/A |
|  | Adaptive questioning | Page 6 |
|  | Number of items | Page 7 |
|  | Completeness check | Page 7 |
|  | Review step | Page 6 |
| Response ranges | Unique site visitor | Page 9 |
|  | View rate (Ratio of unique survey visitors/unique site visitors) | Page 9 |
|  | Participation rate (Ratio of unique visitors who agreed to participate/unique first survey page visitors) | Page 9 |
|  | Completion rate (Ratio of users who finished the survey/users who agreed to participate) | Page 9 |
| Preventing multiple entries from the same individual | Cookies used | Page 9 |
|  | IP check | Page 9 |
|  | Log file analysis | Page 9 |
|  | Registration | Page 5 |
| Analysis | Handling of incomplete questionnaires | Page 9 |
|  | Questionnaires submitted with an atypical timestamp | Page 9 |
|  | Statistical correction | Page 7 to 9 |

## **Supplementary Table 1: Languages spoken by patient populations treated**

| **Patient population, languages spoken** (n, %) | **Health professionals** (n=126) |
| --- | --- |
| English | 124 (98%) |
| Arabic | 19 (15%) |
| Cantonese | 21 (17%) |
| Dari | 7 (6%) |
| Farsi | 19 (15%) |
| Greek | 18 (14%) |
| Hakha Chin | 2 (2%) |
| Hindi | 13 (10%) |
| Italian | 27 (21%) |
| Karen | 4 (3%) |
| Khmer | 2 (2%) |
| Korean | 4 (3%) |
| Kurdish | 1 (1%) |
| Mandarin | 26 (21%) |
| Nepali | 11 (9%) |
| Punjabi | 11 (9%) |
| Sinhalese | 1 (1%) |
| Spanish | 9 (7%) |
| Tagalog | 4 (3%) |
| Thai | 4 (3%) |
| Traditional Chinese | 13 (10%) |
| Turkish | 5 (4%) |
| Vietnamese | 19 (15%) |
| Other^†^ | 6 (5%) |

^†^Other responses reported in Supplementary Table 2

## **Supplementary Table 2: Content analysis of open text survey responses**

| **Survey Question** | **Categorisation of survey response** | **Number of responses** | **% of responses** |
| --- | --- | --- | --- |
| **Please describe your clinical speciality.** | **Physiotherapy clinical speciality (if applicable)** | 5 |  |
|  | Aquatic | 1 | 20% |
|  | Education | 1 | 20% |
|  | Intensive care | 1 | 20% |
|  | Surgical | 1 | 20% |
|  | Emergency | 1 | 20% |
| **Please specify your professional background.** | **Professional background of health professional** | 19 |  |
|  | Neurosurgeon | 1 | 5% |
|  | Paediatric Intensivist | 1 | 5% |
|  | Sports Scientist | 1 | 5% |
|  | Ambulance officer/Paramedic | 2 | 11% |
|  | Retrieval Consultant | 1 | 5% |
|  | Allied Health Assistant | 1 | 5% |
|  | Personal Trainer, Sports Trainer (adolescents) | 1 | 5% |
|  | Pharmacists | 2 | 11% |
|  | Chiropractor | 2 | 11% |
|  | Podiatrist | 1 | 5% |
|  | Dentist | 1 | 5% |
|  | Behaviour Support Practitioner/Counsellor | 1 | 5% |
|  | Pathology | 1 | 5% |
|  | Intensive Care Specialist, Pre-hospital and Retrieval | 1 | 5% |
|  | Exercise Scientist | 1 | 5% |
|  | Chiropractor, Registered Music Therapist | 1 | 5% |
| **Please describe, what sector you work in?** | **Sector worked in** | 12 |  |
|  | Not for profit | 3 | 25% |
|  | Professional sport | 1 | 8% |
|  | Tertiary education/University/Academia/Research | 8 | 67% |
| **Please describe any other languages of patient groups you work with.** | **Languages of patient groups worked with by health professional** | 11 |  |
|  | Bhutanese | 1 | 9% |
|  | Aboriginal languages | 2 | 18% |
|  | Macedonian | 2 | 18% |
|  | Croatian | 2 | 18% |
|  | Serbian | 2 | 18% |
|  | East Timorese | 2 | 18% |
| **Please specify, any other ways that people can sustain a concussion.** | **Other ways people can sustain concussion** | 7 |  |
|  | Recreational activities | 1 | 14% |
|  | Blunt trauma/direct impact to the head | 3 | 43% |
|  | Household accidents | 1 | 14% |
|  | Blast/explosion | 1 | 14% |
|  | Strangulation/choking/suffocation | 1 | 14% |
| **Please specify, any other symptoms of concussion.** | **Other symptoms of concussion** | 32 |  |
|  | Reduced level of consciousness (LOC) | 1 | 3% |
|  | Visual disturbances or problems | 4 | 13% |
|  | Confusion | 3 | 9% |
|  | Mood problems (e.g. suppressed mood, depression) | 3 | 9% |
|  | Balance problems | 1 | 3% |
|  | Autonomic dysfunction | 1 | 3% |
|  | Amnesia / memory issues / recall difficulties | 4 | 13% |
|  | Emotional sensitivity / anger | 2 | 6% |
|  | Thermoregulation | 1 | 3% |
|  | Personality changes | 1 | 3% |
|  | Behaviour changes | 1 | 3% |
|  | Speech changes | 1 | 3% |
|  | Tinnitus | 1 | 3% |
|  | Disorientation | 1 | 3% |
|  | Seizures | 1 | 3% |
|  | Co-ordination difficulties | 1 | 3% |
|  | Fluid and blood from ears | 1 | 3% |
|  | Noise sensitivity | 1 | 3% |
|  | Fatigue | 1 | 3% |
|  | Attention difficulties | 1 | 3% |
|  | Vomiting/nausea | 1 | 3% |
| **Please specify, any other examinations findings that makes you suspect a patient has sustained a concussion?** | **Other examinations findings that make the health professional suspect a person has sustained a concussion** | 9 |  |
|  | Increased reaction time | 1 | 11% |
|  | Impaired dual/multi-tasking ability | 1 | 11% |
|  | Magnetic Resonance Imaging (MRI) | 1 | 11% |
|  | Functional issue/s (e.g. Reading left to right sides) | 2 | 22% |
|  | Gait disturbance | 1 | 11% |
|  | Difference in blood pressure | 1 | 11% |
|  | Reading left to right sides | 1 | 11% |
|  | Spreading reflex | 1 | 11% |
| **Please describe, why you do not use published guidelines to direct your clinical decisions.** | **Reasons why published guidelines are not used to direct health professionals' clinical decisions** | 1 |  |
|  | Concussion not in clinical caseload | 1 | 100% |
| **Please specify, what other published guidelines (not limited to concussion specific guidelines) direct your clinical decisions** | **Other published guidelines (not limited to concussion specific guidelines) that direct health professionals’ clinical decisions** | 21 |  |
|  | Standardised tools for evaluation (e.g. SCAT-6, SCOAT-6) | 1 | 5% |
|  | ACI initial Management of closed head injuries 2nd edition | 1 | 5% |
|  | The Management of Sport Related Concussion in Australian Football 2024 | 1 | 5% |
|  | World Rugby concussion policy | 1 | 5% |
|  | Olympic Winter Institute of Australia Concussion Policy | 1 | 5% |
|  | United States Olympic & Paralympic Committee Concussion Guidelines | 1 | 5% |
|  | Netball Australia Concussion Policy | 2 | 10% |
|  | St John Ambulance Guidelines | 1 | 5% |
|  | AFL Guidelines for SRC | 1 | 5% |
|  | CISG consensus statement on Concussion in Sport (Amsterdam 2022) | 3 | 14% |
|  | Murdoch Children's Research Institute (e.g. Website & headcheck app) | 1 | 5% |
|  | Rugby Australia | 1 | 5% |
|  | Australian OA hip and Knee guidelines | 1 | 5% |
|  | Carpa Manual | 1 | 5% |
|  | Ambulance Victoria Guidelines | 2 | 10% |
|  | Ottawa Living with Concussion Guidelines | 1 | 5% |
|  | England Football learning | 1 | 5% |
| **What websites do you refer to?** | **Websites referred to for concussion resources** | 58 |  |
|  | Children's hospital / RCH | 6 | 10% |
|  | SCAT 5 / SCAT-6 | 2 | 3% |
|  | Australian Institute of Sport (AIS) | 6 | 10% |
|  | Agency for Clinical Innovation (ACI) | 2 | 3% |
|  | Clinicians Health Channel | 1 | 2% |
|  | AFL | 2 | 3% |
|  | Concussion Alliance | 1 | 2% |
|  | Academy of Neurologic Physiotherapy | 1 | 2% |
|  | Google Scholar | 1 | 2% |
|  | Complete Concussion Management Institute | 1 | 2% |
|  | Medscape | 1 | 2% |
|  | Physiopedia | 1 | 2% |
|  | Murdoch Children's Research Institute | 1 | 2% |
|  | Databases/search engines (e.g Ebsco, Pubmed) | 4 | 7% |
|  | Medical speciality site | 1 | 2% |
|  | Connectivity | 3 | 5% |
|  | UpToDate | 2 | 3% |
|  | Clinical Knowledge Network | 1 | 2% |
|  | St John Ambulance Australia clinical resources | 1 | 2% |
|  | Sports Medicine Australia | 3 | 5% |
|  | Queensland Brain Institute | 1 | 2% |
|  | Australian Government Concussion in Sport | 3 | 5% |
|  | ANZ Concussion Guidelines 2024 | 2 | 3% |
|  | It Left No Marks for Health Providers | 1 | 2% |
|  | Raising Children | 1 | 2% |
|  | GP weekly | 1 | 2% |
|  | Medscape | 1 | 2% |
|  | RACGP | 1 | 2% |
|  | Safer Care Victoria | 1 | 2% |
|  | Brain Injury Australia | 1 | 2% |
|  | Synapse | 2 | 3% |
|  | Brain Foundation | 1 | 2% |
|  | Concussion Ontario | 1 | 2% |
| **Please specify, what other health professional(s) do you refer to for diagnosis?** | **Other health professionals referred to for diagnosis** | 3 |  |
|  | Trauma Specialist | 1 | 33% |
|  | Paramedic | 1 | 33% |
|  | Neurosurgeon | 1 | 33% |
| **Please specify, what other health professional(s) do you refer to for treatment?** | **Other health professionals referred to for treatment (if health professional didn't manage the person with a concussion)** | 1 |  |
|  | Concussion Clinic (MDT) | 1 | 100% |
| **Please describe, how frequently would you review/treat a patient with a concussion.** | **Frequency health professionals review/treat a patient with concussion** | 8 |  |
|  | Initial review, and follow up as part of comprehensive geriatric assessment | 1 | 13% |
|  | Walk in only, no scheduled appointments | 1 | 13% |
|  | Monthly, due to fully booked clinic despite reviews needing to be earlier | 1 | 13% |
|  | Acute/subacute phase - weekly | 1 | 13% |
|  | Post concussion syndrome - fortnightly initially, then monthly | 1 | 13% |
|  | Inpatient - daily | 1 | 13% |
|  | Depends on presentation / severity / time from incident | 2 | 25% |
| **Please specify, what other health professional(s) do you refer to for treatment?** | **Other health professionals referred to for treatment (different to their own profession)** | 6 |  |
|  | Concussion specialist multidisciplinary team / Concussion Clinic multidisciplinary team | 2 | 33% |
|  | GP to refer to other health professionals | 1 | 17% |
|  | Naturopath | 1 | 17% |
|  | Specialist Balance/Vestibular clinic | 1 | 17% |
|  | Specific head injury outpatient clinic | 1 | 17% |
| **What other diagnostic images do you request? Please describe.** | **Other diagnostic images requested by health professional** | 1 |  |
|  | None specified (depends on clinical profile +/- imaging performed already) | 1 | 100% |
| **What concussion-specific questionnaires/assessment(s) do you complete?** | **Concussion-specific questionnaires/assessment(s) used by health professional** | 29 |  |
|  | Bespoke neuropsychology interview and assessment | 1 | 3% |
|  | Rivermead Post-Concussion Questionnaire | 9 | 31% |
|  | Concussion Clinical Profile Screen | 2 | 7% |
|  | Graded symptom checklist | 1 | 3% |
|  | Red flags' assessment | 1 | 3% |
|  | Upper Limb Neurological testing | 1 | 3% |
|  | Cranial nerve test | 1 | 3% |
|  | Pronator drift test | 1 | 3% |
|  | Cerebellar testing | 1 | 3% |
|  | Post-Concussion Symptom Scale | 4 | 14% |
|  | Sport Concussion Office Assessment Tool (any version) | 2 | 7% |
|  | Physical assessment | 1 | 3% |
|  | Neurological assessment | 1 | 3% |
|  | Westmead Post Traumatic Amnesia Scale | 1 | 3% |
|  | Cognitive assessment | 1 | 3% |
|  | Functional assessment | 1 | 3% |
| **What symptom-based questionnaires/assessment(s) do you complete?** | **Symptom-based questionnaires/assessment(s) used by health professional** | 24 |  |
|  | Clinical assessment | 1 | 4% |
|  | Rivermead Post-Concussion Questionnaire | 1 | 4% |
|  | Depression, Anxiety and Stress Scale | 3 | 13% |
|  | Post-Traumatic Stress Disorder Checklist | 1 | 4% |
|  | Concussion Clinical Profile Screen | 1 | 4% |
|  | Graded symptom checklist | 1 | 4% |
|  | Dizziness Handicap Inventory | 3 | 13% |
|  | Activities-specific Balance Confidence Scale | 1 | 4% |
|  | Head Injury Assessment and Symptom Scale | 1 | 4% |
|  | Pittsburg Sleep Quality Index | 1 | 4% |
|  | Patient Health Questionnaire | 2 | 8% |
|  | Neck Disability Index | 2 | 8% |
|  | Epworth Sleepiness Scale | 1 | 4% |
|  | Visual Vertigo Analogue Scale | 1 | 4% |
|  | Migraine Disability Assessment Scale | 1 | 4% |
|  | Concussion, Observation, Documentation, Assessment, and Support | 1 | 4% |
|  | Kessler Psychological distress scale K10 | 1 | 4% |
|  | Self-report clinical measure to assess psychiatric symptoms | 1 | 4% |
| **What computerised neurocognitive assessment(s) do you complete?** | **Computerised neurocognitive assessment(s) used by health professional** | 2 |  |
|  | Cogsport/Cognigram | 1 | 50% |
|  | Concussion Tracker app | 1 | 50% |
| **What cognitive assessment(s) do you complete?** | **Cognitive assessment(s) used by health professional** | 28 |  |
|  | Neurological 'checks' in the emergency department | 1 | 4% |
|  | Amnesia scale (not specified) / Post traumatic amnesia testing | 3 | 11% |
|  | Functional cognitive assessments | 1 | 4% |
|  | Westmead Post-Traumatic Amnesia Scale / Abbreviated Westmead Post-Traumatic Amnesia Scale | 7 | 25% |
|  | Montreal Cognitive Assessment | 2 | 7% |
|  | Orientation questions | 1 | 4% |
|  | Orientation log | 1 | 4% |
|  | Post-Concussion Symptom Scale | 1 | 4% |
|  | Neuropsychology assessment | 1 | 4% |
|  | World Health Organisation Disability Assessment Schedule | 1 | 4% |
|  | Glasgow Coma Scale | 2 | 7% |
|  | Cognitive Assessment of Minnesota | 2 | 7% |
|  | Oxford Cognitive Screen (OCS) | 1 | 4% |
|  | Addenbrooke's Cognitive Examination-III | 1 | 4% |
|  | Mini Mental Status Examination | 1 | 4% |
|  | Neuropsychiatry unit cognitive screening tool | 1 | 4% |
|  | Workplace developed 'non-standardised' screens | 1 | 4% |
| **What assessment(s) are required by your health service for you to complete?** | **Assessment(s) required by the health professionals' health service to complete** | 6 |  |
|  | Sport Concussion Assessment Tool 6 | 1 | 17% |
|  | Westmead Post-Traumatic Amnesia Scale | 1 | 17% |
|  | Health service developed assessments (e.g. combined initial assessments) | 2 | 33% |
|  | Kessler Psychological distress scale K10 | 1 | 17% |
|  | Balance outcome measure (not specified) | 1 | 17% |
| **Please specify, any other assessments you complete.** | **Other assessments used by health professionals** | 6 |  |
|  | Neurological observations | 1 | 17% |
|  | Clinical assessment | 1 | 17% |
|  | Assessments as directed by St John Ambulance Clinical Guidelines | 1 | 17% |
|  | Comprehensive chiropractor assessment | 1 | 17% |
|  | Functional assessment | 2 | 33% |
| **Please specify, other assessment you use for ongoing management and monitoring of concussion.** | **Other assessments used by health professionals for ongoing management and monitoring of concussion** | 15 |  |
|  | Balance measures (not specified) | 1 | 7% |
|  | Clinical progress/observations (e.g. progress with vestibular rehab and exercise tolerance, neurological observations) | 2 | 13% |
|  | Buffalo Concussion Treadmill Test | 1 | 7% |
|  | Vestibular assessments (BPPV, head thrust) | 1 | 7% |
|  | Vestibular and ocular motor screening (VOMS) | 3 | 20% |
|  | Vertebrobasilar Insufficiency testing | 1 | 7% |
|  | Cervical assessment | 1 | 7% |
|  | Cervicogenic dizziness assessment | 1 | 7% |
|  | HeadCheck App | 1 | 7% |
|  | Chiropractor assessment | 1 | 7% |
|  | Functional assessment | 2 | 13% |
| **What kind of medical specialist do you refer people with concussion to where symptoms don't resolve within 4 weeks?** | **Other medical specialist health professionals refer people with concussion to where symptoms don't resolve within 4 weeks** | 76 |  |
|  | Rehabilitation physician/specialist | 6 | 8% |
|  | Sports physician/specialist | 8 | 11% |
|  | GP | 20 | 26% |
|  | Neurologist | 20 | 26% |
|  | Concussion Clinic | 3 | 4% |
|  | Emergency department / ED doctor or specialist / Hospital | 6 | 8% |
|  | Physician (not specified) | 1 | 1% |
|  | Neuro-ophthalmologist | 1 | 1% |
|  | Neuro-otologist | 1 | 1% |
|  | Orthopaedics | 1 | 1% |
|  | Cardiologist | 1 | 1% |
|  | Psychiatrist | 1 | 1% |
|  | Multi-disciplinary service / Specialist clinic (not specified) | 2 | 3% |
|  | Neuropsychologist | 2 | 3% |
|  | ABI service | 1 | 1% |
|  | Psychologist | 1 | 1% |
|  | Geriatrician | 1 | 1% |
| **Please specify, how frequently you review a patient as part of close observation?** | **Frequency health professional reviews a person as part of close observation** | 3 |  |
|  | Twice weekly | 1 | 33% |
|  | Daily | 1 | 33% |
|  | Pro re nata (PRN) | 1 | 33% |
| **What else do you do to manage those people with concussion where symptoms don't resolve within 4 weeks? Please describe.** | **Other ways health professionals manage people with concussion where symptoms don’t resolve within 4 weeks** | 5 |  |
|  | Outside scope of practice, onward referral (not specified) | 1 | 20% |
|  | Referral to rehabilitation doctor | 1 | 20% |
|  | Referral to GP for onward specialist referral | 1 | 20% |
|  | Referral to other allied health | 1 | 20% |
|  | Specific intervention approaches (e.g. education, fatigue management, activity recommendations) | 1 | 20% |
|  | Private clinic (specialist vestibular rehabilitation) | 1 | 20% |
| **Please list what websites you refer your patients to.** | Websites health professionals refer their patients with concussion | 13 |  |
|  | AIS (e.g. concussion page) | 3 | 23% |
|  | Concussion Alliance | 1 | 8% |
|  | YouTube, e.g. for BPPV treatment | 1 | 8% |
|  | Raising children | 1 | 8% |
|  | Headcheck app information | 1 | 8% |
|  | Murdoch Children's Research Institute | 1 | 8% |
|  | Health Direct | 1 | 8% |
|  | Google | 1 | 8% |
|  | Netball Australia Guidelines | 1 | 8% |
|  | State Health website (e.g. Royal Children's Hospital for children, Queensland Health for adults) | 1 | 8% |
|  | Connectivity | 1 | 8% |
| **Please describe what other resources you give your patients.** | **Other concussion resources provided to patients by health professionals** | 10 |  |
|  | Youtube videos | 1 | 10% |
|  | Referral to outpatient clinics | 1 | 10% |
|  | HeadCheck app | 2 | 20% |
|  | AFL community guidelines | 1 | 10% |
|  | SCAT-6 | 1 | 10% |
|  | Internationally developed patient handouts (e.g. Canadian) | 1 | 10% |
|  | Handouts (e.g. stages of concussion, pacing activities, sleep) | 2 | 20% |
|  | Verbal instructions (e.g. seek GP advice if symptoms not resolved) | 1 | 10% |
|  | Service specific resources | 5 | 50% |
| **Please describe what other differential diagnoses you consider when considering concussion (considering acute, subacute or chronic conditions).** | **Other differential diagnoses (acute, subacute or chronic conditions) considered when considering concussion** | 13 |  |
|  | Vestibular injury /dysregulation | 1 | 8% |
|  | BPPV | 2 | 15% |
|  | Cervical spine injury | 1 | 8% |
|  | Neck, ear or eye injury | 1 | 8% |
|  | Functional Neurologic Disorder | 1 | 8% |
|  | Persistent Postural-Perceptual Dizziness | 1 | 8% |
|  | Chronic fatigue | 1 | 8% |
|  | ADHD | 1 | 8% |
|  | Autism | 1 | 8% |
|  | Depression | 1 | 8% |
|  | Functional neurological fatigue | 1 | 8% |
|  | Previous cognitive impairment | 1 | 8% |
| **What other features do you think could indicate the possible presence of CTE? Please describe.** | **Other features health professionals think could indicate the possible presence of CTE** | 4 |  |
|  | Cognitive and functional decline | 1 | 25% |
|  | History of frequent head trauma | 1 | 25% |
|  | History of frequent concussions of suspected concussions | 1 | 25% |
|  | Balance disorders | 1 | 25% |
| **Please describe what other populations are thought to be at risk of developing CTE?** | **Other populations are thought to be at risk of developing CTE** | 7 |  |
|  | Multiple head injuries/concussions (e.g. motor vehicle accidents) | 3 | 43% |
|  | Equestrians | 1 | 14% |
|  | Contact sports | 1 | 14% |
|  | Amateur boxers | 1 | 14% |
|  | Younger people who engage in risky behaviours (e.g. that involve head knocks, whip lash injuries) | 1 | 14% |

## **Supplementary Table 3: Frequency of concussion being a secondary diagnosis in clinical practice**

| **Frequency of concussion being a secondary diagnosis in clinical practice,** (n, %) | **Health professionals**  (n=126)^¶^ |
| --- | --- |
| Never | 14 (11%) |
| Rarely | 54 (43%) |
| About half the time | 13 (10%) |
| Frequently | 9 (7%) |
| ‘Not applicable, this is not something I do within my current scope of practice’ | 36 (29%) |

## **Supplementary Table 4: Health professional’ knowledge of chronic traumatic encephalopathy (CTE)**

|  | Health professionals  (n, %) |
| --- | --- |
| **Health professional knowledge of chronic traumatic encephalopathy (CTE)** |  |
| No knowledge | 37 (29%) |
| Somewhat familiar | 35 (28%) |
| Moderately familiar | 29 (23%) |
| Familiar | 17 (14%) |
| Very familiar | 3 (2%) |
| Health profession indicates that they have heard of CTE, and are familiar with the research but don’t believe that there is enough research to support individuals being diagnosed with CTE | 5 (4%) |
| **Health professional knowledge of the association of CTE with repeated head injuries** (*“True or False: CTE has been associated with repeated head injuries”*) |  |
| True | 91 (72%) |
| False | 2 (2%) |
| Unsure | 33 (26%) |
| **Health professional knowledge of patient populations at risk of developing CTE** *(responded ‘yes’)* |  |
| Victims of Domestic/Family Violence | 110 (89%) |
| Athletes | 116 (94%) |
| Military populations | 88 (71%) |
| Other^†^ | 9 (7%) |

^†^ ’Other’ responses reported in Supplementary Table 2

## **Supplementary Figure 1: Health professional knowledge of symptoms of CTE**

|  | % of participants correctly responded | % of participants incorrectly responded |
| --- | --- | --- |
| Motor features (e.g. gait and balance, dysarthria) | 56% | 44% |
| Mood/affect or other psychiatric features (e.g. depression) | 58% | 42% |
| Behavioural features (e.g. impulsiving, agitation) | 63% | 37% |
| Cognitive features (e.g. memory loss, confusion, executive functioning difficulties) | 67% | 33% |
